# An evaluation roadmap for critical quality attributes from tier 1 in analytical similarity assessment

**DOI:** 10.1371/journal.pone.0208354

**Published:** 2018-12-06

**Authors:** Kejian Wu, Haitao Pan, Chen Li, Qingbo Zhao, Ling Wang, Jielai Xia

**Affiliations:** 1 Department of Health Statistics, Fourth Military Medical University, Xi’an, Shannxi, China; 2 Department of Mathematics and Physics, Fourth Military Medical University, Xi’an, Shannxi, China; 3 Department of Biostatistics, St. Jude Children’s Research Hospital, Memphis, Tennessee, United States of America; Harbin Medical University, CHINA

## Abstract

Analytical similarity assessment of critical quality attributes (CQAs) serves as a foundation for the development of biosimilar products and facilitates an abbreviated subsequent clinical evaluation. In this study, we establish a statistical evaluation roadmap with statistical approaches for some selected CQAs from Tier 1, because they are most relevant to clinical outcomes and require the most rigorous statistical methods. In the roadmap, we incorporate 3 methods—ranking and tier assignment of quality attributes, the equivalence test, and the Mann–Whitney test for equivalence—that are important to determine analytical similarity between the reference and biosimilar products. For the equivalence test, we develop a power calculation formula based on the two one-sided tests procedure. Exact sample sizes can be numerically calculated. Then, we propose a flexible idea for selecting the number of reference lots (*n*_*R*_) and the number of biosimilar lots (*n*_*T*_) to adjust for serious unbalanced sample sizes. From results of extensive simulations under various parameter settings, we obtain a workable strategy to determine the optimum sample size combination (*n*_*T*_, *n*_*R*_) for the equivalence test of CQAs from Tier 1. R codes are provided to facilitate implementation of the roadmap and corresponding methods in practice.

## Introduction

Biosimilars are biological products that are highly similar but not identical to their reference products, notwithstanding minor differences in clinically inactive components. Thus, biosimilars are close but not exact copies of biological products that are already on the market. With the expiration of patents on many innovative biological products, biosimilar products have received increasing attention from pharmaceutical companies such as Celltrion [1], Pfizer [2], and Sandoz [3] and from regulatory agencies such as the European Medicines Agency [4], United States Food and Drug Administration (FDA) [5, 6], World Health Organization [7], and China Food and Drug Administration [8]. Biosimilars can offer affordable treatment alternatives for diseases such as cancer and chronic inflammatory disorders.

**Fig 1:**
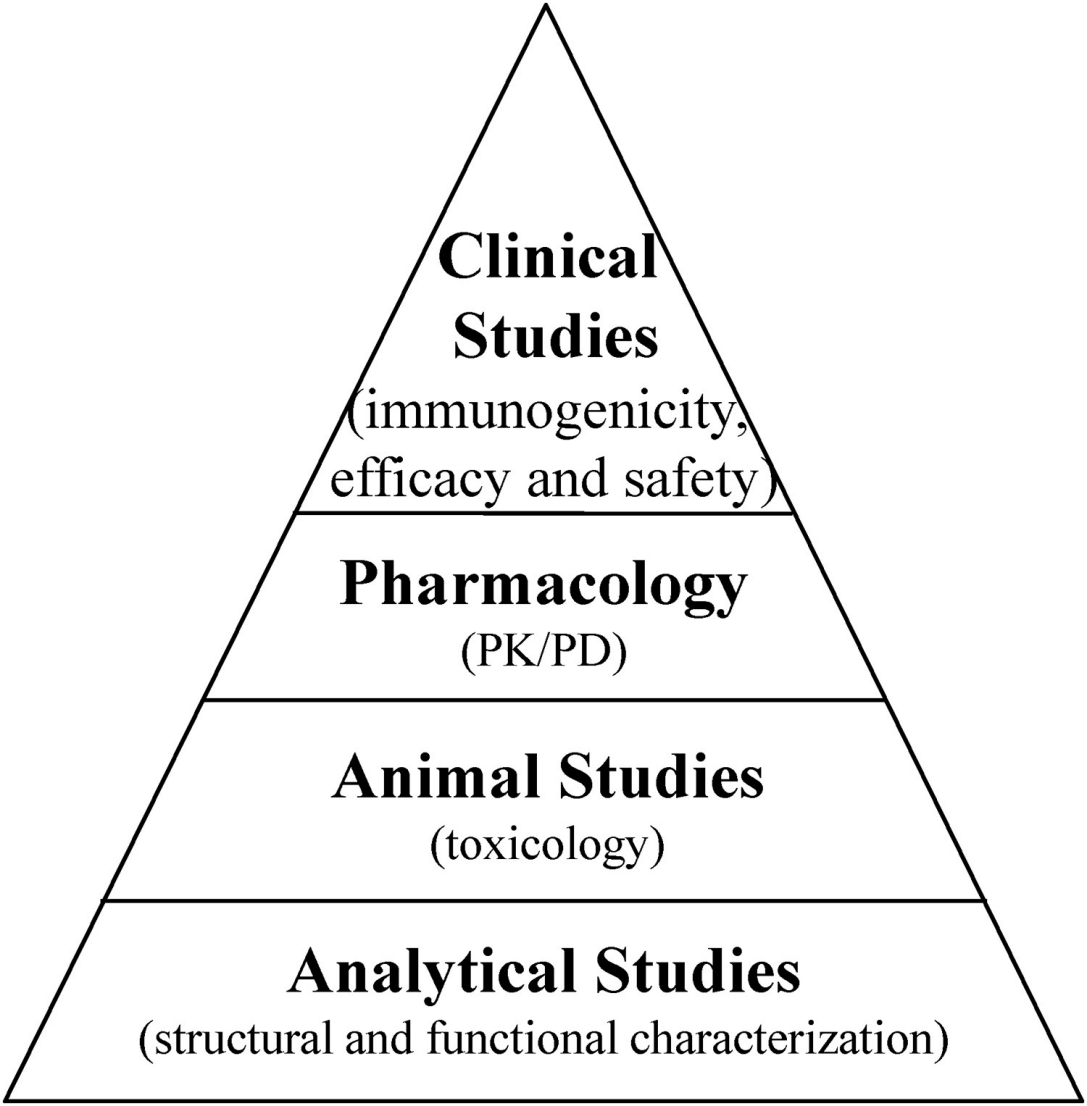
Stepwise approach to assess biosimilarity. PK: pharmacokinetics; PD: pharmacodynamics.

It is important for biosimilar developers to understand how to demonstrate that the product is biosimilar to its reference product. FDA guidelines recommend a stepwise approach to generate data needed to demonstrate biosimilarity [5]. The stepwise approach is briefly summarized in the pyramid, as shown in Fig 1 proposed by Chow [9]. The stepwise approach starts with analytical studies of critical quality attributes (CQAs) that are relevant to clinical outcomes [10]. The shape of the pyramid signifies that fewer data are required in the clinical phase if adequate biosimilarity has been established in previous steps. For example, comprehensive analytical characterization was used to assess the analytical similarity between ABP 501 and 2 adalimumab products [11] and between ABP 215 and both United States–and European Union–sourced bevacizumab products [12].

Considering that there may be a large number of CQAs in practice, Chow [9] and Tsong et al. [10] proposed a statistical approach for demonstrating analytical similarity based on a tiered system that accounts for their criticality, for example, most (Tier 1), mild to moderate (Tier 2), and least (Tier 3) relevant to clinical outcomes. They also recommended the equivalence test of means for CQAs from Tier 1, the quality range approach for CQAs from Tier 2, and visual displays for CQAs from Tier 3. Since the most rigorous statistical method is required for CQAs from Tier 1, many statisticians have performed important pioneering studies on CQAs from Tier 1. For example, Chow et al. discussed properties of the equivalence test [13], justification for margin [14], and sample size [15]. Tsong et al. provided details of the equivalence test [10]. Dong et al. proposed 2 sample size imbalance adjustment methods [16]. Other issues have been considered in Shen et al. [17], Burdick et al. [18], Dong et al. [19], Chen et al. [20], Liao et al. [21], and Wu et al. [22].

However, these studies have often focused on a particular statistical issue and have not developed a complete evaluation system for biosimilar developers, especially those conducting quality analytical tests. Therefore, in this study, we develop a statistical evaluation roadmap for some selected CQAs from Tier 1, focusing on both statistical methods and simplicity of implementation. The goal of our roadmap is to provide evaluation procedures to biosimilar developers in an accessible manner.

This paper is organized as follows. Section 2 introduces key factors in the evaluation roadmap: (i) the risk ranking and tier assignment of quality attributes (QAs), (ii) statistical considerations of equivalence test—power function and sample size required, and (iii) Mann–Whitney test for equivalence for seriously skewed analytical data. Section 3 presents a case study. Section 4 presents concluding remarks with discussions.

## Methods

For analytical similarity assessment of a biosimilar, a comprehensive analytical characterization is performed to compare the proposed biosimilar and reference products. For physical/chemical characterization of products, we can obtain a large number of testing values of QAs by using state-of-the-art analytical methods. These QAs may include general properties, primary structure, higher-order structure, particles and aggregates, product-related substances and impurities, biological activity and forced thermal degradation, and so on. It is impractical to statistically compare all QAs to demonstrate biosimilarity. Thus, the identification of CQAs among QAs is an important first step in analytical similarity assessment, which is based on a thorough understanding of the potential for QAs to affect safety and efficacy. Thus, we first introduce a systematic scientific and risk-based approach to identify CQAs and assign their tiers. Second, we study statistical approaches for the equivalence test for some selected CQAs from Tier 1. Successful completion of these steps will ensure that there is sufficient evidence to demonstrate that a proposed biosimilar is highly similar to its reference product in analytical similarity assessment.

### Ranking and tier assignment of quality attributes

To identify CQAs from a lot of QAs, we recommend the risk ranking and filtering approach developed by Roche/Genentech [23]. This approach focuses on drug safety and efficacy and incorporates 2 factors: impact and uncertainty of that impact. Impact is assigned on a 2- to 20-point scale that considers the known or potential effect of an attribute on 4 clinical performance categories: bioactivity, pharmacokinetics, immunogenicity, and safety. Uncertainty is based on the confidence that biosimilar developers have in the relevance of the information used in impact assessment. Uncertainty is assigned on a 1- to 7-point scale, with lower scores reflecting higher confidence. Then, the risk score of an attribute is generated by multiplying the 2 values of impact and uncertainty:

Risk=Impact(2−20)×Uncertainty(1−7). (1)

The highest risk score of the above 4 categories is used to categorize the QA as CQA or non-CQA. Then, 13 risk scores are selected as the cutoff. That is, attributes having risk scores greater than 13 in any single impact category are classified as CQAs. Alt et al. provide further details on the ranking and determination of CQAs and examples from monoclonal antibodies [23].

After many QAs are classified as CQAs, biosimilar developers need to determine the appropriate tier of CQAs. Tiers are assigned based on the risk score, and Tier 1 is reserved for the highest risk scores that have a direct impact on clinical outcomes. In addition to the highest risk scores, several other factors such as quantitative or qualitative data and the level of assays used for assessing attributes should also be considered [24]. Criticality and determination of tiering of CQAs are assessed mainly by biosimilar developers in the analytical characterization or biocharacterization team. In the following subsections, we propose statistical approaches for some selected CQAs from Tier 1 that are appropriate for the equivalence test.

### Equivalence test for CQAs from Tier 1

We conduct the test for equivalence of means of selected CQAs from Tier 1 between the proposed biosimilar and reference products. Let *T* and *R* be the responses of a given CQA from Tier 1 for the biosimilar (or test) product and its reference product, respectively. Assuming that *T* and *R* follow a $N(\mu_{T},\sigma_{T}^{2})$ and $N(\mu_{R},\sigma_{R}^{2})$ distribution, where *μ*_*T*_ and *μ*_*R*_ are mean values, $\sigma_{T}^{2}$ and $\sigma_{R}^{2}$ are the variances, respectively. By using a parallel design, we test the following hypothesis:

$$H_{0}:\mu_{T}-\mu_{R}\leq -\delta \;\;\text{or}\;\;\mu_{T}-\mu_{R}\geq \delta \;\;\text{vs}.\;H_{a}:-\delta <\mu_{T}-\mu_{R}<\delta ,$$ (2)

where δ > 0 is the equivalence margin. This type of test can be decomposed into Schuirmann’s two one-sided tests [25], in which *H*_0_ and *H*_*a*_ in (2) are tested separately by a one-sided test:

$$H_{01}:\mu_{T}-\mu_{R}\leq -\delta \text{vs}.H_{a1}:\mu_{T}-\mu_{R}>-\delta$$ (3)

$$H_{02}:\mu_{T}-\mu_{R}\geq \delta \;\;\;\text{vs}.\;H_{a2}:\mu_{T}-\mu_{R}<\delta .$$ (4)

We then reject *H*_01_ at the *α* level of significance in (3) if

$$T_{L}=\frac{({\bar{X}}_{T}-{\bar{X}}_{R})+\delta }{\sqrt{S_{T}^{2}/n_{T}+S_{R}^{2}/n_{R}}}>t_{\alpha ,\nu }$$ (5)

and reject *H*_02_ in (4) if

$$T_{U}=\frac{({\bar{X}}_{T}-{\bar{X}}_{R})-\delta }{\sqrt{S_{T}^{2}/n_{T}+S_{R}^{2}/n_{R}}}<-t_{\alpha ,\nu },$$ (6)

where sample sizes *n*_*T*_ and *n*_*R*_ refer to the number of lots from the proposed biosimilar and the reference product required in the equivalence test, respectively. ${\bar{X}}_{T},{\bar{X}}_{R}$ and *S*_*T*_, *S*_*R*_ are the sample mean and standard deviation (SD) of the proposed biosimilar and the reference products, respectively. The symbol *t*_*α*,*v*_ is the *α* 100%th percentile of the *t*-distribution with the degrees of freedom approximated by Satterthwaite’s approximation as $\nu ={\left(S_{T}^{2}/n_{T}+S_{R}^{2}/n_{R}\right)}^{2}/\left[S_{T}^{4}/\left[n_{T}^{2}(n_{T}-1)\right]+S_{R}^{4}/\left[n_{R}^{2}(n_{R}-1)\right]\right]$ [26].

The global null hypothesis *H*_0_ in (2) is rejected with type I error *α* if both one-sided hypotheses (3) and (4) are rejected with type I error *α*. Thus, we conclude that there is sufficiently strong evidence to support statistical equivalence in means if both one-sided hypotheses *H*_01_ in (3) and *H*_02_ in (4) are rejected.

An alternative method to assess similarity between the 2 products is to use a two-sided confidence interval (CI) for *μ*_*T*_ − *μ*_*R*_. We conclude that there is statistical equivalence in means if the 100(1 − 2*α*)% CI $\left({\bar{X}}_{T}-{\bar{X}}_{R}-t_{\alpha ,\nu }\cdot \sqrt{\frac{S_{T}^{2}}{n_{T}}+\frac{S_{R}^{2}}{n_{R}}},{\bar{X}}_{T}-{\bar{X}}_{R}+t_{\alpha ,\nu }\cdot \sqrt{\frac{S_{T}^{2}}{n_{T}}+\frac{S_{R}^{2}}{n_{R}}}\right)$ for *μ*_*T*_ − *μ*_*R*_ lies within the interval (−δ, δ).

#### Power function of the equivalence test

In this section, we derive the power function of the statistical test to test the hypotheses in (2). We need to consider determining the proper equivalence margin δ first, which is the critical and challenging step in the equivalence test. In this paper, on the basis of previous studies such as those by Chow [9], Tsong et al. [10], and others, we take the equivalence margin δ as a function of the variability of the reference product with the form of δ = *f* × *σ*_*R*_, where *f* is a constant. The variability *σ*_*R*_ is unavailable to the biosimilar developer and is conventionally estimated by sample SD of the reference product. The multiplier *f* can be adjusted by the pre-given power 1 − *β* and the true underlying mean difference between the proposed biosimilar and reference products. Here, the true underlying mean difference is denoted by *μ*_*T*_ − *μ*_*R*_ = θ and it is also considered as a function of *σ*_*R*_, i.e., *μ*_*T*_ − *μ*_*R*_ = θ = *η* × *σ*_*R*_, where *η* is a prespecified tolerable shift. Differences in population mean are expected between biosimilar and reference products, because biosimilar products made from living cells or organisms have a much larger variability than do generic drug products. Thus, the equivalence test allows a mean shift of *η* × *σ*_*R*_ and the target mean difference is *μ*_*T*_ − *μ*_*R*_ = *η* × *σ*_*R*_.

Under a parallel design and the hypothesis (2), since the $\frac{\nu (S_{T}^{2}/n_{T}+S_{R}^{2}/n_{R})}{\sigma_{T}^{2}/n_{T}+\sigma_{R}^{2}/n_{R}}$ approximately follows a chi-squared distribution with *v* degrees of freedom based on the Welch–Satterthwaite equation [27], the exact power function can be derived by modifying the power formula for the crossover bioequivalence study proposed by Shen et al. [28]:

$$\begin{array}{l} \text{power}=P\left(\theta ,n_{T},n_{R},\sigma_{T}^{2},\sigma_{R}^{2}\right)\\ =P\left\{\text{Reject}\;H_{0}|\theta =\mu_{T}-\mu_{R},\theta \in (-\delta ,\delta ),\sigma_{T}^{2},\sigma_{R}^{2}\right\}\\ =P\left\{\frac{-\delta -\theta }{\sqrt{S_{T}^{2}/n_{T}+S_{R}^{2}/n_{R}}}+t_{\alpha ,\nu }<\frac{({\bar{X}}_{T}-{\bar{X}}_{R})-\theta }{\sqrt{S_{T}^{2}/n_{T}+S_{R}^{2}/n_{R}}}<\frac{\delta -\theta }{\sqrt{S_{T}^{2}/n_{T}+S_{R}^{2}/n_{R}}}-t_{\alpha ,\nu }|\theta \in (-\delta ,\delta ),\sigma_{T}^{2},\sigma_{R}^{2}\right\}\\ =\int_{0}^{A}\{\Phi \left(\frac{\delta -\theta }{\sqrt{\sigma_{T}^{2}/n_{T}+\sigma_{R}^{2}/n_{R}}}-t_{\alpha ,\nu }\sqrt{\frac{x}{\nu }}\right)-\Phi \left(\frac{-\delta -\theta }{\sqrt{\sigma_{T}^{2}/n_{T}+\sigma_{R}^{2}/n_{R}}}+t_{\alpha ,\nu }\sqrt{\frac{x}{\nu }}\right)\}\cdot f(x)\text{d}x, \end{array}$$ (7)

where Φ(·) is the standard normal cumulative distribution function and *f*(*x*), the probability density function of the chi-squared distribution, can be written as $f(x)=\frac{1}{2^{\nu /2}\Gamma (\nu /2)}x^{\nu /2-1}e^{-x/2}$. The upper limit of the integral is defined as $A=\frac{\nu \cdot \delta^{2}}{t_{\alpha ,\nu }^{2}\cdot \left(\sigma_{T}^{2}/n_{T}+\sigma_{R}^{2}/n_{R}\right)}$. Formula (7) can be adapted for the equivalence test with equal and unequal variance. We can calculate power values and determine the sample size for the equivalence test in analytical similarity assessment from (7) by using a standard numerical integration. It should be noted that the sample size formula in analytical studies for similarity assessment proposed by Chow et al. [15] is given by $n_{T}=\frac{{\left(z_{\alpha }+z_{\beta /2}\right)}^{2}\sigma^{2}(1+1/k)}{{\left(\delta -\left|\mu_{T}-\mu_{R}\right|\right)}^{2}}$ assuming that $\sigma_{R}^{2}=\sigma_{T}^{2}=\sigma^{2}$, where *k* = *n*_*T*_/*n*_*R*_ and *z*_*α*_ is the upper *α* quantile of the standard normal distribution (for example, *z*_0.05_ = 1.645). The sample size formula by Chow et al. should be obtained based on the approximate power:

$$\Phi \left(\frac{\delta -\theta }{\sigma \sqrt{1/n_{T}+1/n_{R}}}-z_{\alpha }\right)+\Phi \left(\frac{\delta +\theta }{\sigma \sqrt{1/n_{T}+1/n_{R}}}-z_{\alpha }\right)-1\approx 2\Phi \left(\frac{\delta -\theta }{\sigma \sqrt{1/n_{T}+1/n_{R}}}-z_{\alpha }\right)-1.$$ (8)

The above approximate power formula (8) works very well when the sample size is large. It may underestimate the power if the sample size is too small. Therefore, we prefer the explicit formula (7) for sample size determination and various simulation studies.

Using formula (7), we conducted several simulation studies under various parameter settings, including different *f* and *η*, sample sizes (*n*_*T*_, *n*_*R*_), and ratios of variances $\sigma_{R}^{2}/\sigma_{T}^{2}$. The simulation of various parameter settings is necessary. For example, we may need to increase the constant *f* when sample reference variability may be underestimated if reference values are correlated because of the same source. Under the assumption that $\sigma_{T}^{2}=\sigma_{R}^{2}$, S1 and S2 Files provide details of simulation results. S1 File lists the assigned power for different values of the multiplier *f* (from 1 to 2.5 by 0.02) and the given number of lots per product *n* (from 3 to 25 by 1) with *μ*_*T*_ − *μ*_*R*_ = 1/8 × *σ*_*R*_ and *α* = 0.05. S2 File gives results of the assigned power for cases of different *η* values (from 1/16 to 1/2 by 1/16) and the given number of lots per product *n* (from 3 to 25 by 1) with *f* = 1.5 and *α* = 0.05. Note that when we choose the equivalence margin as δ = 1.5 × *σ*_*R*_ and the true mean difference as *μ*_*T*_ − *μ*_*R*_ = 1/8 × *σ*_*R*_, *n*_*T*_ = *n*_*R*_ = 9 are required to achieve an 80% power at the 5% level of significance. That is, 9 biosimilar and reference lots are sufficient to make meaningful comparisons. Furthermore, the test has 87% power to reject the null hypothesis in favor of equivalence when *n*_*T*_ = *n*_*R*_ = 10 with equal variance.

#### Sample size requirement

**Fig 2:**
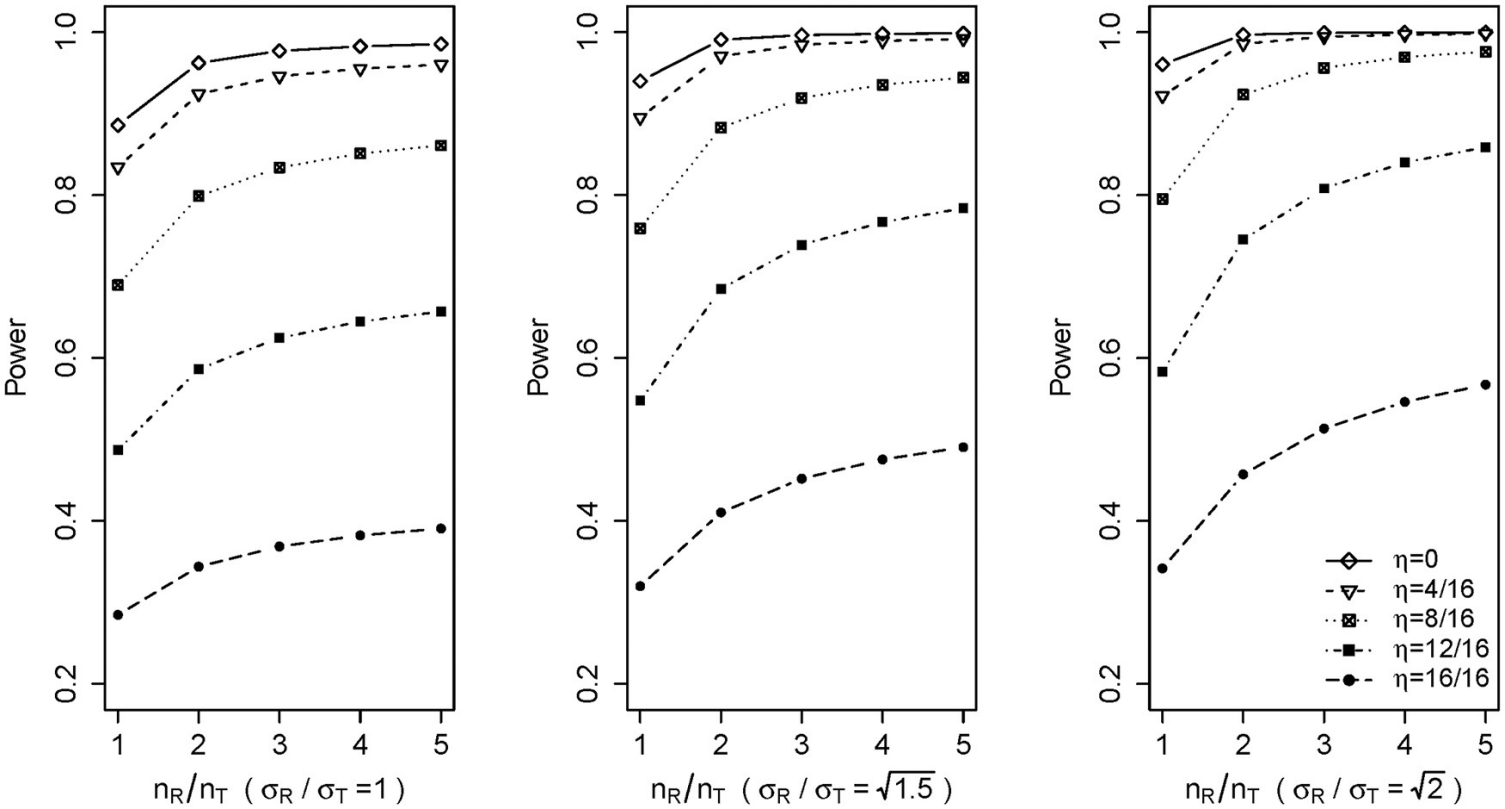
Power with *n*_*T*_ = 10 and margin δ = 1.5 × *σ*_*R*_ at different values of the sample size ratio, variance ratio, and true mean difference.

Another commonly encountered question is how to handle large sample size imbalance in determining the number of reference lots and the number of test lots required in the equivalence test. As is often the case, the available reference lots denoted by *N*_*R*_ are usually larger than the available biosimilar lots denoted by *N*_*T*_, because biosimilar developers need a sufficient number of reference lots to understand the reference product. Directly choosing *n*_*T*_ = *N*_*T*_ and *n*_*R*_ = *N*_*R*_ in the above equivalence test may lead to concerns that the information of the reference product can potentially dominate the power of the equivalence test [16]. We can conduct a simulation study to compare power to explain why sample size imbalance needs to be adjusted using formula (7). In Fig 2, we give an example for simulation results for *n*_*T*_ = 10. For each *n*_*T*_, *n*_*R*_ increases from *n*_*R*_ = *n*_*T*_ to *n*_*R*_ = *5n*_*T*_ and 3 ratios of variances *σ*_*R*_/*σ*_*T*_ are chosen: 1, $\sqrt{1.5}$, and $\sqrt{2}$. The multiplier *η* in the true mean difference between the biosimilar and reference products, *μ*_*T*_ − *μ*_*R*_ = *η* × *σ*_*R*_, increases from 0 to 1. Fig 2 shows that a biosimilar product with a larger mean difference *μ*_*T*_ − *μ*_*R*_ can achieve the desired power by increasing the sample size of 1 arm *n*_*R*_ only. For example, when *σ*_*R*_/*σ*_*T*_ = 1, *η* = 8/16, and *n*_*T*_ = *n*_*R*_ = 10, we can increase the power of the equivalence test from about 70% to above 80% by only increasing *n*_*R*_ to 50. To avoid the case in which a large mean difference may be overlooked, we need to adjust sample size imbalance to make *n*_*T*_ ≤ *n*_*R*_ ≤ 1.5*n*_*T*_.

Chow et al. [15] also proposed that sample size imbalance can be adjusted by the appropriate *λ* in the relationship *n*_*R*_ = *λ* × *n*_*T*_. However, both the reference and test lots are often very limited and the coefficient *λ* is often a decimal and difficult to determine. Thus, we establish a more flexible relationship between the *n*_*R*_ and *n*_*T*_ required as *n*_*R*_ = *n*_*T*_ + *k* in the equivalence test, where *k* = 0,1,…,⌈0.5*n*_*T*_⌉; the symbol "⌈ ⌉" returns the value of a number rounded upward to the nearest integer. The proposed relationship can guarantee that *n*_*R*_ is within [*n*_*T*_,1.5*n*_*T*_] and nearly balanced with *n*_*T*_, even for sample sizes as small as 10. On the basis of the above relationship and the power function presented in formula (7), we can calculate the minimum *n*_*T*_ for various selections of *k* in the simulation study. Once the mininum *n*_*T*_ has been determined, we can determine the values of *k* and *n*_*R*_ required in the equivalence test.

Table 1 gives examples of simulation results for 1 − *β* = 80%, 85%, and 90% when *f* = 1.5 (equivalence margin δ = 1.5 × *σ*_*R*_) and *η* = 1/8 (true underlying mean difference *μ*_*T*_ − *μ*_*R*_ = 1/8 × *σ*_*R*_) with *σ*_*R*_ = *σ*_*T*_. From Table 1, it is easy to determine that the minimum *n*_*T*_ = 8 and choose *k* = 2 to satisfy the relationship *n*_*R*_ ∈ [*n*_*T*_, 1.5*n*_*T*_], that is, (*n*_*T*_, *n*_*R*_) = (8,10) to achieve an 80% power at the 5% level of significance in an equivalence test for CQAs from Tier 1. The combinations (*n*_*T*_, *n*_*R*_) = (7,11),(7,12) do not meet the criterion of *n*_*R*_ being within [*n*_*T*_, 1.5*n*_*T*_]. Obviously, there are many other alternative combinations of sample sizes, such as (*n*_*T*_, *n*_*R*_) = (9,9), (9,10), and (8,11). The reason for taking (*n*_*T*_, *n*_*R*_) = (8,10) as the optimum combination is that it can ensure the lowest number of sample sizes for biosimilar products. Similarly, the optimum combination is (8,12) for a nominal 85% power and (10,12) for a nominal 90% power.

**Table 1:** Sample size *n*_*T*_ for different *k* values and powers for *α* = 0.05 with *σ*_*R*_ = *σ*_*T*_.

Power(1–β)	*k* = 0	*k* = 1	*k* = 2	*k* = 3	*k* = 4	*k* = 5
80%	9	9	8	8	7^a^	7^a^
85%	10	10	9	9	8	8^a^
90%	11	11	10	10	10	9^a^

^a^ Value does not meet the criterion that *n*_*R*_ is within[*n*_*T*_, 1.5*n*_*T*_].

For different *f* (from 1 to 2.5 by 0.02) and *η* (from 1/16 to 1/2 by 1/16) values, the optimum combination (*n*_*T*_, *n*_*R*_) with *α* = 0.05, 1 − *β* = 80%, 85%, and 90% under the assumption of *σ*_*R*_ = *σ*_*T*_ are shown in S3 File. Hence, the optimum combinations given first minimize the number of biosimilar lots and then determine *n*_*R*_ based on an appropriate *k*. Note that if there are enough biosimilar lots *N*_*T*_, equal sample sizes are preferred to assess analytical similarity, such as (*n*_*T*_, *n*_*R*_) = (9,9), (10,10), and (11,11) for achieving 1 − *β* = 80%, 85%, and 90%, respectively.

After *n*_*R*_ has been determined on the basis of the above simulation result, *n*_*R*_ needs to be randomly selected from the available reference lots *N*_*R*_. When selecting *n*_*R*_ from *N*_*R*_, to reduce the sampling error associated with simple random samples, different *n*_*R*_ lots should be chosen through simulation studies with at least 100,000 replications to determine whether a high proportion (e.g., >80% of these replications) yields the same results in the equivalence test. In practice, we use the entire available reference lots *N*_*R*_ to estimate *σ*_*R*_ to establish the equivalence margin δ = *f* × *σ*_*R*_.

### Mann–Whitney test for equivalence

The above discussion demonstrates that the sample size in the equivalence test for CQAs from Tier 1 is relatively small. In this situation, the assumption of normality for data may be violated, and a distribution-free or nonparametric test may be more appropriate for comparing these independent samples. We consider using the Mann–Whitney test for equivalence, which is sensitive to divergences between any 2 continuous distributions. For simplicity, let *T*_*i*_ and *R*_*j*_ be observations of the biosimilar and reference arms. If the 2 distributions of *T*_*i*_ and *R*_*j*_ are equivalent, then the probability that any value of *T*_*i*_ is greater than any value of *R*_*j*_ denoted by *π*_+_ = *P*[*T*_*i*_ > *R*_*j*_] should be approximately 1/2. Alternatively, the null hypothesis is that *π*_+_ is either smaller or larger than the range of equivalence. Therefore, the Mann–Whitney test for equivalence uses a rank-sum statistic to test whether *π*_+_ is within the small range of approximately 1/2. Thus, the equivalence hypothesis for the non-parametric test of testing problem (2) is given by

$$H_{0}:\pi_{+}\leq 1/2-\delta^{\prime }\;\text{or}\;\pi_{+}\geq 1/2+\delta^{\prime }\;\text{vs}.\;H_{a}:1/2-\delta^{\prime }<\pi_{+}<1/2+\delta^{\prime },$$ (9)

where δ′ is defined by $\delta^{\prime }=\Phi (\delta /\sqrt{2\sigma^{2}})-1/2$, where *σ* is the pooled standard deviation of *T*_*i*_ and *R*_*j*_. The value *π*_+_ is estimated using the Mann–Whitney statistic, and the estimator *W*_+_ defined as $W_{+}=1/\left(n_{T}n_{R}\right)\sum_{i=1}^{n_{T}}\sum_{j=1}^{n_{R}}I(T_{i}-R_{j})$ is given with the indicator of a positive sign $I(x)=\{\begin{array}{cc} 1 & \text{for}\;x>0\\ 0 & \text{for}\;x\leq 0 \end{array}$.

Rejecting the nonequivalence *H*_0_ in (9) if and only if

$$\left|W_{+}-1/2\right|/{\hat{\sigma }}_{W_{+}}<C(\alpha ,\delta^{\prime }),$$ (10)

where

$${\hat{\sigma }}_{W_{+}}^{2}=\frac{1}{n_{T}n_{R}}\left(W_{+}-(n_{T}+n_{R}-1)\;W_{+}^{2}+(n_{T}-1)\prod_{XXY}+(n_{R}-1)\;\prod_{XYY}\right),$$

$$\prod_{XXY}=\frac{2}{n_{T}n_{R}(n_{T}-1)}\sum_{i_{1}=1}^{n_{T}-1}\sum_{i_{2}=i_{1}+1}^{n_{T}}\sum_{j=1}^{n_{R}}I(T_{i_{1}}-R_{j})\cdot I(T_{i_{2}}-R_{j}),$$

and

$$\prod_{XYY}=\frac{2}{n_{T}n_{R}(n_{R}-1)}\sum_{i=1}^{n_{T}}\sum_{j_{1}=1}^{n_{R}-1}\sum_{j_{2}=j_{1}+1}^{n_{R}}I(T_{i}-R_{j_{1}})\cdot I(T_{i}-R_{j_{2}}),$$

and *C*^2^(*α*, δ′) is the *α* 100%th percentile of the non-central chi-squared distribution with degrees of freedom equal to 1 and positive noncentrality parameters equal to ${\delta^{\prime }}^{2}/{\hat{\sigma }}_{W_{+}}^{2}$. The Mann–Whitney test for equivalence is asymptotically distribution free with respect to the significance level and controls the level even for sample sizes as small as 10. Details of the derivation process of formulas and the calculation method have been rigorously established by Wellek [29].

So far, we have developed an analytical similarity evaluation roadmap that includes our proposed statistical approaches for CQAs from Tier 1. Key steps of the roadmap are described as follows:

*Step 1*: Determine the CQAs from Tier 1 through the systematic risk ranking and tiering approach we introduced.
*Step 2*: Determine the margin as given in S1–S3 Files, select *n*_*T*_, *k*, *n*_*R*_, and then determine the sample size.
*Step 3*: Conduct the equivalence test or Mann–Whitney test for equivalence for CQAs of interest from Tier 1 and draw relevant conclusions.

## Case study

In this case study, we have acquired the analytical data for 2 CQAs from a pharmaceutical company, to show how our proposed statistical evaluation roadmap can be used to assess analytical similarity. Because of the commercial confidentiality, sensitive information such as the name of the CQA is masked and data are used only as examples to validate the methods for both equivalence test and Mann-Whitney test.

**Fig 3:**
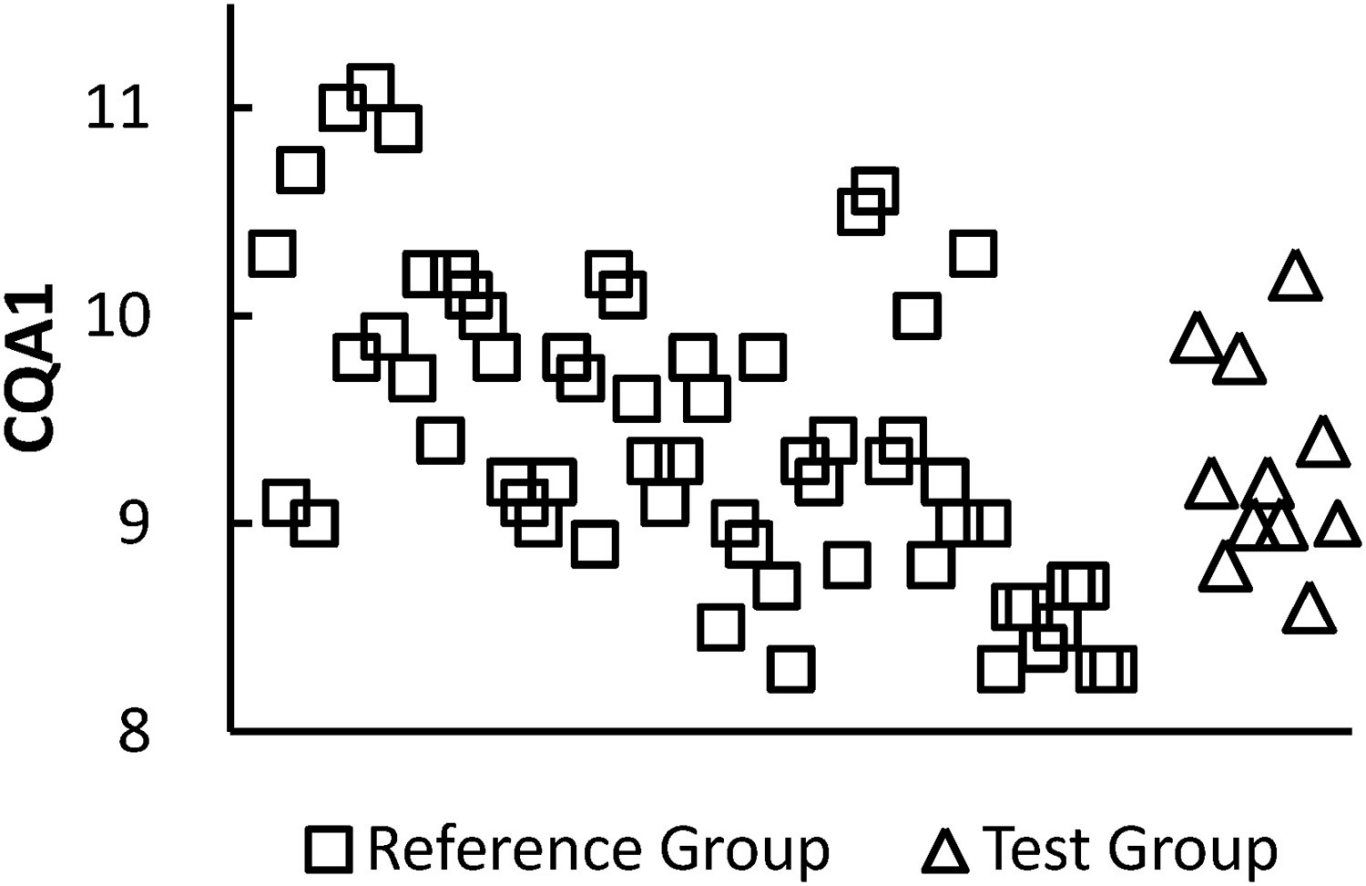
Analytical data for CQA1 from each lot. CQA: critical quality attribute.

**Fig 4:**
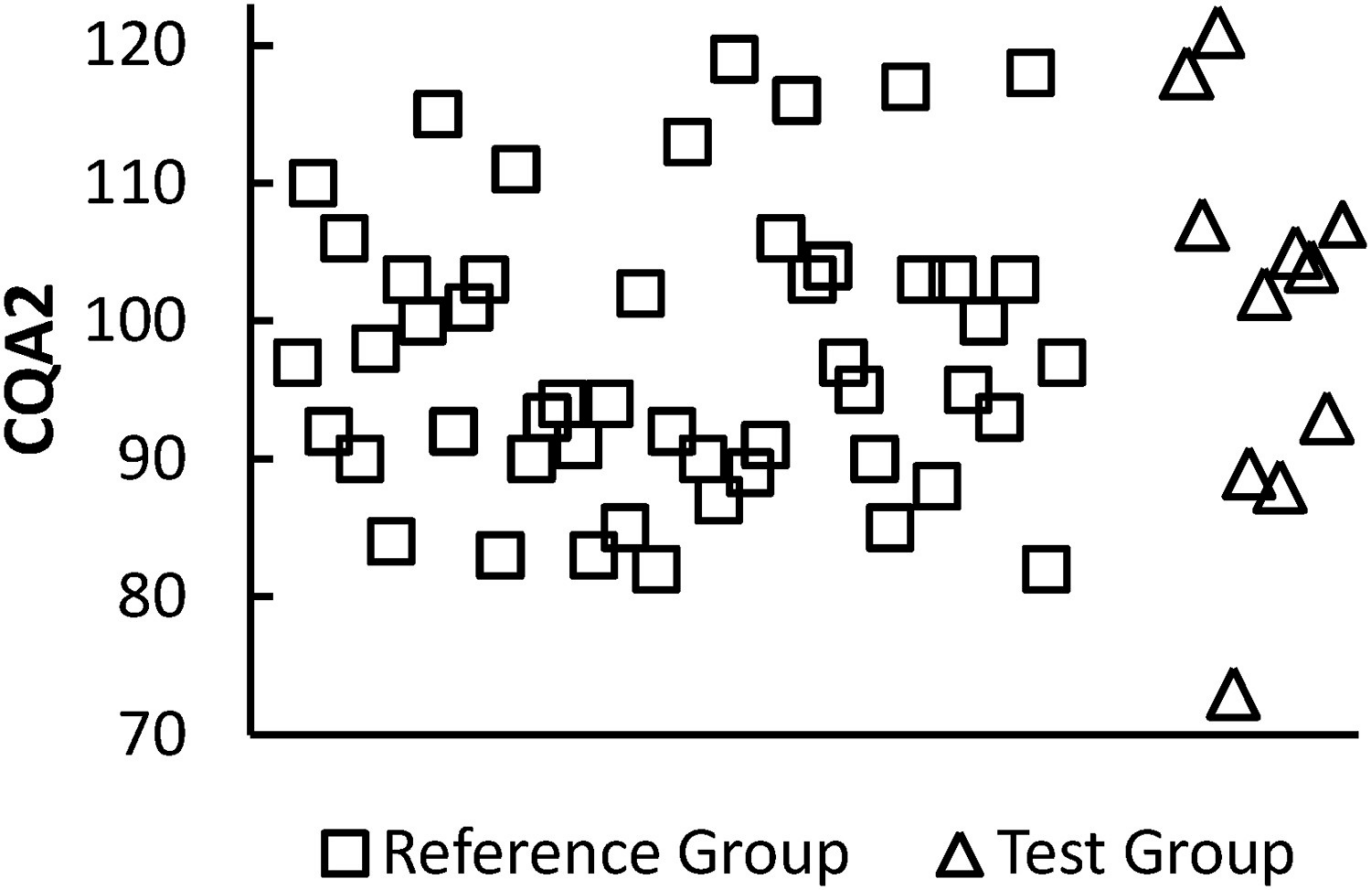
Analytical data for CQA2 from each lot. CQA: critical quality attribute.

The 2 CQAs have been identified by relevant company, especially researchers in the quality control team, and based on the risk ranking and tier assignment approach that we previously introduced. Numerical values are assigned to impact and uncertainty and multiplied to generate a relative risk score. Finally, the 2 CQAs having the highest risk ranking among attributes and are suited for statistical tests are considered the most relevant to clinical outcomes assigned to Tier 1 after a rigorous internal discussion among drug developers. S4 File gives analytical data for CQA1 and CQA2 of the reference and test groups. Analytical data include 11 lots of the test group and 61 lots of the reference group for CQA1, and 11 lots of the test group and 50 lots of the reference group for CQA2. Analytical data of 2 CQAs from each lot are shown in Figs 3 and 4, respectively. Both figures show large overlaps between the test and reference groups. It is clear that the sample size for the reference group, denoted by *N*_*R*_, is larger than that for the test group, denoted by *N*_*T*_, that is, *N*_*R*_ ≫ *N*_*T*_. Table 2 shows summary statistics for the 2 CQAs.

**Table 2:** Summary statistics for CQA1 and CQA2.

Statistics	CQA1		CQA2	
	RG	TG	RG	TG
Number of lots	61	11	50	11
Mean	9.46	9.28	97.50	100.64
SD	0.78	0.50	10.15	13.95
%CV	8.28	5.34	10.41	13.86
*P-*value^a^	0.049	0.428	0.048	0.705

CQA: critical quality attribute; RG: reference group; TG: test group; SD: standard deviation; CV: coefficient of variation.

^a^ *P-*values were calculated for the Shapiro–Wilk normality test.

Using CQA1 as an example, we can perform a similar analysis for CQA2. Table 3 summarizes the parameter settings and results of statistical evaluation. First, CQA1 undergoes the statistical equivalence test. To compare the reference and test groups, sufficient communication is needed with drug developers. Then, the multiplier *f* = 1.5 for the margin δ = *f* × *σ*_*R*_ and the multiplier *η* = 1/8 for the true underlying mean difference *μ*_*T*_ − *μ*_*R*_ = 1/8 × *σ*_*R*_ is determined. Since *N*_*R*_ is much larger than *N*_*T*_ in CQA1, it is not appropriate to directly make *n*_*T*_ = *N*_*T*_ and *n*_*R*_ = *N*_*R*_ in the equivalence test and it is necessary to make some adjustments for imbalanced sample size. We first determine that *n*_*T*_ = *N*_*T*_ = 11 and then divide the reference lots *N*_*R*_ into 2 parts according the *n*_*R*_ required. As shown in S1 File, under δ = 1.5 × *σ*_*R*_ and *μ*_*T*_ − *μ*_*R*_ = 1/8 × *σ*_*R*_, the power achieved is nearly 91% at the 5% level of significance when the sample size is 11 for both the groups. Hence, we choose *k* = 0 and make *n*_*R*_ = *n*_*T*_ + *k* = 11. To establish the equivalence margin δ, we use the entire available reference lots *N*_*R*_ to estimate *σ*_*R*_. Consequently, we obtain (*n*_*T*_, *n*_*R*_) = (11, 11) and margin = (–1.17,1.17) in the equivalence test for CQA1. As shown in Table 3, the high proportion (97.66%) of CI of 10^5^ random samples is completely within the margin (–1.17,1.17) for CQA1. Here, we also list results of the Mann–Whitney test for equivalence with (*n*_*T*_, *n*_*R*_) = (11, 11) and margin = (0.13,0.87). The Mann–Whitney test could lose power when the normality assumption for data is valid. In this case study, we claim that the CQA1 of 2 groups is analytically similar, based on results of the equivalence test, because the analytical data are approximately normally distributed. If the analytical data have a seriously skewed distribution, we will make a decision based on results of the Mann–Whitney test.

**Table 3:** Summarized results of statistical evaluation for CQA1 and CQA2.

Test of conduct	Parameter	CQA1	CQA2
Equivalence test of means	Equivalence margin ^a^	(–1.17,1.17)	(–15.23,15.23)
	Sample sizes (*n*_*T*_, *n*_*R*_)	(11, 11)	(11, 11)
	Random samples	10^5^	10^5^
	Proportion	97.66%	88.83%
Conclusion: Analytically similar		Yes	Yes
Mann–Whitney test for equivalence	Equivalence margin ^b^	(0.13,0.87)	(0.16,0.84)
	Sample sizes (*n*_*T*_, *n*_*R*_)	(11, 11)	(11, 11)
	Random samples	10^5^	10^5^
	Proportion	93.60%	73.19%

CQA, critical quality attribute.

^a^ Margin of the equivalence test is $\left(-1.5{\hat{\sigma }}_{R},1.5{\hat{\sigma }}_{R}\right)$.

^b^ Margin of the Mann–Whitney test is $\left(1-\Phi (1.5{\hat{\sigma }}_{R}/\sqrt{2\sigma^{2}}),\Phi (1.5{\hat{\sigma }}_{R}/\sqrt{2\sigma^{2}})\right)$.

In summary, statistical evaluations for the 2 CQAs demonstrate the analytical similarity between the reference and test groups. R programs are provided in S5 File for readers to get detailed results using the proposed methods, including the equivalence test and the Mann–Whitney test for equivalence.

## Conclusions

We propose a statistical evaluation roadmap using feasible statistical methods for analytical similarity assessment of CQAs from Tier 1. The statistical evaluation roadmap has 3 advantages: (i) there is a very flexible relationship between *n*_*R*_ and *n*_*T*_, as *n*_*R*_ = *n*_*T*_ + *k* in the equivalence test; (ii) there is much more flexibility in choosing parameters such as equivalence margins and the true underlying mean difference as well as in obtaining optimum sample sizes; and (iii) the Mann–Whitney test is used for analytical data that follow a skewed distribution. Using this roadmap, we found sufficiently strong evidence to support the similarity between the reference and biosimilar products. A sufficient degree of biosimilarity demonstrated in the earlier step of head-to-head analytical assessment can serve as a foundation to develop biosimilars and facilitate an abbreviated subsequent preclinical and clinical evaluation, thus enabling a shorter path to licensing. This is different from the typical development of a new small-molecule drug, wherein the pathway heavily focuses on the endpoints of clinical evaluations relating to demonstrating efficacy and safety in humans.

Although there are several advantages of the proposed roadmap, there are still some unsolved issues. First, the variability of the reference is underestimated when the method does not consider the case in which we sample more than one item from each lot, which leads to a conservative test and affects sample size determination [30]. Second, when the available reference lots *N*_*R*_ are larger than the available biosimilar lots *N*_*T*_, the *n*_*R*_ lots required in the equivalence test need to be randomly selected from *N*_*R*_. Thus, the *N*_*R*_ lots are divided into 2 parts: *n*_*R*_ and *N*_*R*_ − *n*_*R*_. We use the entire data of *N*_*R*_ lots to estimate *σ*_*R*_ to establish the equivalence margin in our evaluation roadmap. Further discussion is required for the case in which the first part contains the *n*_*R*_ lots or the second part contains the remaining reference sample *N*_*R*_ − *n*_*R*_ lots used to determine the equivalence margin. Our future studies will focus on incorporating these challenges into the current proposed framework.

## Supporting information

S1 File Assigned power for *f* and *n*. (XLS)

S2 File Assigned power for *η* and *n*. (XLS)

S3 File Optimum combination of sample size. (XLSX)

S4 File Analytical testing value of CQA1 and CQA2. (XLSX)

S5 File R programs. (DOC)
