# Supplementary material for: An evaluation roadmap for critical quality attributes from tier 1 in analytical similarity assessment
Source: PLoS One. 2018 Dec 6;13(12):e0208354. doi: 10.1371/journal.pone.0208354 (PMC6283468; doi:10.1371/journal.pone.0208354)
Supplement: S5 File — (DOC) [file pone.0208354.s005.doc]

**Formal statistical tests of equivalence of the CQAs from Tier1**

#-------------------------------------------Input-----------------------------------------------------#

# xT--> the analytical data for the test products #

# xR--> the analytical data for the reference product #

# f --> The multiplier f in equivalence margin delta #

# delta is regarded as a function with delta = f *sigmaR #

# nsample --> The number that nR are randomly selected from the NR #

# eta --> The multiplier eta in the true difference muT-muR=eta* sigmaR #

# --------------------------------------------------------------------------------------------------------#

##################################################################

# In "EquivalenceTest1", we use the entire available reference lots NR to estimate sigmaR #

# Here sigmaR is the standard deviation of the CQA of the reference product #

EquivalenceTest1<- function(xT, xR, f, nsample, eta=1/8, alpha=0.05){

deltaU=f*sd(xR)

deltaL=-f*sd(xR)

theta=eta*sd(xR)

count=0

for (i in 1:nsample)

{

nT=length(xT)

reference=sample(xR,nT,replace=F)

nT=length(xT)

nR=length(reference)

ST=sd(xT)

SR=sd(reference)

degree=(ST^2/nT+SR^2/nR)^2/(1/(nT-1)*ST^4/nT^2+1/(nR-1)*SR^4/nR^2)

T1=(mean(xT)-mean(reference))-qt(1-alpha, degree)*sqrt(ST^2/nT+SR^2/nR)

T2=(mean(xT)-mean(reference))+qt(1-alpha, degree)*sqrt(ST^2/nT+SR^2/nR)

if( T1>deltaL && T2<deltaU )

count=count+1

}

proportion=count/nsample

if(proportion>=0.8)

{

cat("Analytical similarity!\n\n")

cat("The equivalence test in means supports a demonstration of analytical similarity in this CQA with a high proportion", proportion,".\n")}

else {

cat("No analytical similarity!\n\n")

cat("The data of this CQA fail the equivalence test depending on the split sample we choose because the propotion is only", proportion,".\n")

}

}

###########################################################

# In "EquivalenceTest2", we use the remaining reference lots(the second split part) NR-nR to estimate sigmaR#

EquivalenceTest2<- function(xT, xR, f, nsample, eta=1/8, alpha=0.05){

count=0

for (i in 1:nsample)

{

nT=length(xT)

reference=sample(xR,nT,replace=F)

nT=length(xT)

nR=length(reference)

ST=sd(xT)

SR=sd(reference)

deltaU=f*sd(setdiff(xR,reference))

deltaL=-f*sd(setdiff(xR,reference))

theta=eta*sd(setdiff(xR,reference))

degree=(ST^2/nT+SR^2/nR)^2/(1/(nT-1)*ST^4/nT^2+1/(nR-1)*SR^4/nR^2)

T1=(mean(xT)-mean(reference))-qt(1-alpha, degree)*sqrt(ST^2/nT+SR^2/nR)

T2=(mean(xT)-mean(reference))+qt(1-alpha, degree)*sqrt(ST^2/nT+SR^2/nR)

if( T1>deltaL && T2<deltaU )

count=count+1

}

proportion=count/nsample

if(proportion>=0.8)

{

cat("Analytical similarity!\n\n")

cat("The equivalence test in means supports a demonstration of analytical similarity in this CQA with a high proportion", proportion,".\n")}

else {

cat("No analytical similarity!\n\n")

cat("The data of this CQA fail the equivalence test depending on the split sample we choose because the propotion is only", proportion,".\n")

}

}

############## Case study for CQA1 #############

set.seed(1)

xT=c(9.9 ,9.2 ,8.8 ,9.8 ,9.0 ,9.2 ,9.0 ,10.2 ,8.6 ,9.4 ,9.0)

xR=c(10.3 ,9.1 ,10.7 ,9.0 ,11.8 ,11.0 ,9.8 ,11.1 ,9.9 ,10.9 ,9.7 ,10.2 ,9.4 ,10.2 ,10.1 ,10.0 ,9.8 ,9.2 ,9.1 ,9.0 ,9.2 ,9.8 ,9.7 ,8.9 ,10.2 ,10.1 ,9.6 ,9.3 ,9.1 ,9.3 ,9.8 ,9.6 ,8.5 ,9.0 ,8.9 ,9.8 ,8.7 ,8.3 ,9.3 ,9.2 ,9.4 ,8.8 ,10.5 ,10.6 ,9.3 ,9.4 ,10.0 ,8.8 ,9.2 ,9.0 ,10.3 ,9.0 ,8.3 ,8.6 ,8.6,8.4,8.5,8.7,8.7,8.3,8.3)

f=1.5

nsample=10^5

EquivalenceTest1(xT, xR, f, nsample)

EquivalenceTest2(xT, xR, f, nsample)

############## Case study for CQA2 #############

set.seed(1)

xT=c(118 ,107 ,121 ,73 ,89 ,102 ,88 ,105 ,104 ,93 ,107 )

xR=c(97 ,110 ,92 ,106 ,90 ,98 ,84 ,103 ,100 ,115 ,92 ,101 ,103 ,83 ,111 ,90 ,93 ,94 ,91 ,83 ,94 ,85 ,102 ,82 ,92 ,113 ,90 ,87 ,119 ,89 ,91 ,106 ,116 ,103 ,104 ,97 ,95 ,90 ,85 ,117 ,103 ,88 ,103 ,95 ,100 ,93 ,103 ,118 ,82 ,97)

f=1.5

nsample=10^5

EquivalenceTest1(xT, xR, f, nsample)

EquivalenceTest2(xT, xR, f, nsample)

**Mann-Whitney test for equivalence of the CQAs from Tier 1**

#-----------------------------------------------Input----------------------------- ------------------------#

# xT--> the analytical data for the test products #

# xR--> the analytical data for the reference product #

# f --> The multiplier f in equivalence margin delta #

# delta is regarded as a function with delta = f *sigmaR #

# nsample --> The number that nR are randomly selected from the NR #

# ------------------------------------------------------------------------------------------------------------#

####################################################################

rm(list=ls())

MWTest = function(xT, xR, f, nsample,alpha=0.05){

count=0

for (i in 1:nsample)

{

nT=length(xT)

x =sample(xR,nT,replace=F)

y =xT

eps1_ = pnorm(f/sqrt(2))-1/2

eps2_ = pnorm(f/sqrt(2))-1/2

m = length(x)

n = length(y)

eqctr = 0.5 + (eps2_-eps1_)/2

eqleng = eps1_ + eps2_

wxy = 0

pihxxy = 0

pihxyy = 0

for (i in 1:m)

for (j in 1:n)

wxy = wxy + trunc(0.5*(sign(x[i] - y[j]) + 1))

for (i in 1:m)

for (j1 in 1:(n-1))

for (j2 in (j1+1):n)

pihxyy = pihxyy + trunc(0.5*(sign(x[i] - max(y[j1],y[j2])) + 1))

for (i1 in 1:(m-1))

for (i2 in (i1+1):m)

for (j in 1:n)

pihxxy = pihxxy + trunc(0.5*(sign(min(x[i1],x[i2]) - y[j]) + 1))

wxy = wxy / (m*n)

pihxxy = pihxxy*2 / (m*(m-1)*n)

pihxyy = pihxyy*2 / (n*(n-1)*m)

sigmah = sqrt((wxy-(m+n-1)*wxy**2+(m-1)*pihxxy+(n-1)*pihxyy)/(m*n))

crit = sqrt(qchisq(alpha,1,(eqleng/2/sigmah)**2))

if (abs((wxy-eqctr)/sigmah) >= crit) rej = 0

if (abs((wxy-eqctr)/sigmah) < crit) rej = 1

if (is.na(sigmah) || is.na(crit)) rej = 0

if( rej==1 )

count=count+1

}

proportion=count/nsample

if(proportion>=0.8)

{

cat("Analytical similarity!\n\n")

cat("The Mann-Whitney test supports a demonstration of analytical similarity in this CQA with a high proportion", proportion,".\n")}

else {

cat("No analytical similarity!\n\n")

cat("The data of this CQA can fail the Mann-Whitney test depending on the split sample we choose because the propotion is only", proportion,".\n")

}

}

############## Case study for CQA1 #############

set.seed(1)

xT=c(9.9 ,9.2 ,8.8 ,9.8 ,9.0 ,9.2 ,9.0 ,10.2 ,8.6 ,9.4 ,9.0)

xR=c(10.3 ,9.1 ,10.7 ,9.0 ,11.8 ,11.0 ,9.8 ,11.1 ,9.9 ,10.9 ,9.7 ,10.2 ,9.4 ,10.2 ,10.1 ,10.0 ,9.8 ,9.2 ,9.1 ,9.0 ,9.2 ,9.8 ,9.7 ,8.9 ,10.2 ,10.1 ,9.6 ,9.3 ,9.1 ,9.3 ,9.8 ,9.6 ,8.5 ,9.0 ,8.9 ,9.8 ,8.7 ,8.3 ,9.3 ,9.2 ,9.4 ,8.8 ,10.5 ,10.6 ,9.3 ,9.4 ,10.0 ,8.8 ,9.2 ,9.0 ,10.3 ,9.0 ,8.3 ,8.6 ,8.6,8.4,8.5,8.7,8.7,8.3,8.3)

f=1.5

SC=sqrt(((length(xT)-1)*var(xT)+(length(xR)-1)*var(xR))/(length(xT)+length(xR)-2))

nsample=10^5

MWTest(xT, xR, f*sd(xR)/SC, nsample)

############## Case study for CQA2 #############

set.seed(1)

xT=c(118 ,107 ,121 ,73 ,89 ,102 ,88 ,105 ,104 ,93 ,107 )

xR=c(97 ,110 ,92 ,106 ,90 ,98 ,84 ,103 ,100 ,115 ,92 ,101 ,103 ,83 ,111 ,90 ,93 ,94 ,91 ,83 ,94 ,85 ,102 ,82 ,92 ,113 ,90 ,87 ,119 ,89 ,91 ,106 ,116 ,103 ,104 ,97 ,95 ,90 ,85 ,117 ,103 ,88 ,103 ,95 ,100 ,93 ,103 ,118 ,82 ,97)

f=1.5

SC=sqrt(((length(xT)-1)*var(xT)+(length(xR)-1)*var(xR))/(length(xT)+length(xR)-2))

nsample=10^5

MWTest(xT, xR, f*sd(xR)/SC, nsample)

**References**

R package: EQUIVNONINF. Available from:

<https://cran.r-project.org/web/packages/EQUIVNONINF/> .Accessed 19 [Sep](../../../../../AppData/Local/Youdao/DictBeta/Application/7.0.1.0214/resultui/dict/result.html) 2017.
